# Inhibition of RAGE Attenuates Cigarette Smoke-Induced Lung Epithelial Cell Damage via RAGE-Mediated Nrf2/DAMP Signaling

**DOI:** 10.3389/fphar.2018.00684

**Published:** 2018-07-02

**Authors:** Hanbyeol Lee, Jooyeon Lee, Seok-Ho Hong, Irfan Rahman, Se-Ran Yang

**Affiliations:** ^1^Department of Thoracic and Cardiovascular Surgery, Kangwon National University, Chuncheon, South Korea; ^2^Department of Internal Medicine, Kangwon National University, Chuncheon, South Korea; ^3^Department of Environmental Medicine, University of Rochester Medical Center, Rochester, NY, United States

**Keywords:** cigarette smoke extract, COPD, FPS-ZM1, RAGE, DAMP, Nrf2

## Abstract

The oxidative stress and cellular apoptosis by environmental factor including cigarette smoke induces alveolar airway remodeling leading to chronic obstructive pulmonary disease (COPD). Recently, the receptor for advanced glycan end products (RAGE) which is highly expressed in alveolar epithelium is emerging as a biomarker for COPD susceptibility or progression. However, it still remains unknown how RAGE plays a role in cigarette smoke extract (CSE)-exposed human alveolar type II epithelial cell line. Therefore, we determined the efficacy of RAGE-specific antagonist FPS-ZM1 in response to CSE-induced lung epithelial cells. CSE induced the elevated generation of RONS and release of pro-inflammatory cytokines, and impaired the cellular antioxidant defense system. Further, CSE induced the alteration of RAGE distribution via the activation of redox-sensitive DAMP (Damage-associated molecular patterns) signaling through Nrf2 in cells. Although pre-treatment with SB202190 (p38 inhibitor) or SP600125 (JNK inhibitor) failed to recover the alteration of RAGE distribution, treatment of FPS-ZM1 significantly exhibited anti-inflammatory and anti-oxidative/nitrosative effects, also inhibited the activation of redox-sensitive DAMP signaling through Nrf2 (nuclear factor erythroid 2-related factor 2) migration in the presence of CSE. Taken together, our data demonstrate that RAGE and Nrf2 play a pivotal role in maintenance of alveolar epithelial integrity.

## Introduction

Cigarette smoke (CS) is a major contributor in reproductive and developmental effects (Boucherat et al., 2016), aging (Sundar et al., 2015), cancer (Stampfli and Anderson, 2009), cardiovascular disease (Morris et al., 2015; Lahousse et al., 2016), and pulmonary diseases including chronic obstructive pulmonary disease (COPD)/emphysema and idiopathic pulmonary fibrosis. In cigarette smoking-related pulmonary diseases, more than 80% of COPD is developed due to CS exposure (Laniado-Laborin, 2009) and higher risk of people suffering from pulmonary diseases are devastating conditions like multiple apoptotic cells in the parenchyma (epithelium) and airways in the lung. Alveolar epithelium is consisting of two main cell types: the alveolar type I and alveolar type II cell. The type II cells comprise only 4% of the alveolar surface area, yet they display a multifunctional pneumocyte including synthesis and secretion of surfactant, xenobiotic metabolism, transepithelial movement of water, progenitor cells capable of self-renewal and differentiation into type I pneumocyte for regeneration of the alveolar epithelium following lung injury (Castranova et al., 1988; Barkauskas et al., 2013). Generally, CS exhaustively causes typical alveolar cells including types I and II epithelial cells to increase chronic inflammation and protease/antiprotease imbalance related to the enormous oxidative stress and free radicals, followed by programmed cell death (Malhotra et al., 2009; Barnes, 2013). In various bioinformatics including microarray, genome wide association studies (GWAS), gene expression profiling, next generation sequencing (NGS) or integrating genomic data, they identified that cultured human epithelial cells from COPD patients or mouse alveolar epithelial cells to CS or CS extract (CSE) induce significant variability of gene relevant to smoking-associated lung disease, particularly emphysema and lung function (DeMeo et al., 2009; Pillai et al., 2009; Siedlinski et al., 2011; Soler Artigas et al., 2011; Hackett et al., 2012; Silverman and Loscalzo, 2012; Zhou et al., 2013; Cho et al., 2014; Hobbs and Hersh, 2014). A considerable number of study proposed that advanced glycosylation end product-specific receptor (*AGER*) is a functional gene associated with ratio for forced expiratory volume in 1 s (FEV1) to forced vital capacity (FVC) (Cheng et al., 2013; Iwamoto et al., 2014; Kim et al., 2014). The protein of *AGER*, the receptor for advanced glycation end products (RAGE), contributes to both chronic inflammatory and tissue remodeling processes (Yan et al., 2010) in many refractory and chronic organ degenerative disease, such as Alzheimer’s disease (Galasko et al., 2014; Walker et al., 2015), Parkinson’s disease, diabetes (Soro-Paavonen et al., 2008), atherosclerosis (Bro et al., 2008; Harja et al., 2008) and chronic lung disease (Nicolls and Laubach, 2014; Oczypok et al., 2017). In mammalian, three major forms of RAGE have been well established. The most studied of the RAGE isoform is full-length RAGE (mRAGE or xRAGE), inducing oxidative stress to stimulate signal transduction including inflammation, chemotaxis and apoptosis depending on specificity of cell type (Schmidt et al., 2000). Otherwise, the truncated circulating soluble form of RAGE undergoing RNA splicing (esRAGE) or proteolysis (sRAGE) lacks the transmembrane domain of full-length RAGE, released into the extracellular space and suppressing the activation of full-length RAGE and intracellular danger signals known as damage-associated molecular patterns (DAMPs) signaling. When these the expression of RAGE isoform and their signaling become aberrantly regulated either by persisting stimuli or by impeded resolution, they turn out to be highly destructive for the surrounding epithelium and may causes severe damage and epithelium break down as observed in chronic obstructive disease/emphysema In our recent study, we showed upregulation of cellular expression of RAGE, initiating inflammatory response, and downregulation of soluble RAGE, acting as a ‘decoy’ in protein, serum and bronchoalveolar lavage fluid in the elastase-induced experimental COPD mice and in cell lysate in CSE-induced mouse alveolar type II epithelial cells as well as in the serum and protein of COPD patients (Lee H. et al., 2017). Additionally, we investigated the efficacy of a RAGE-specific antagonist chemical to combat the life-threatening effects of COPD, and revealed that blockade of RAGE rescued deleterious effects at the molecular, cellular, and tissue levels with downstream DAMPs danger signals *in vivo* and *in vitro*, and reversed emphysematous lung symptoms through DAMP-Nrf2 signaling. The protective role of RAGE blocking in human cells has not been investigated. In this study, our goal is to investigate the mechanistic interactions between epithelial cells and RAGE-DAMP signaling through Nrf2 with particular focus on the potential protective effects of RAGE antagonizing in CSE induced human epithelial cell damage. This results make it a basis for future study for RAGE blockade-based therapies in airways disease.

## Materials and Methods

### Preparation of Cigarette Smoke Extract (CSE)

Cigarette smoke extract was prepared as previously described using impingers by vigorous bubbling from one 3R4F cigarette into 10 ml of serum free-DMEM (Dulbecco’s Modified Eagle Medium). The solution was filtered through a 0.22-um and considered a 10% CSE solution (Lee et al., 2016). CSE was prepared within 30 min and stored at -80°C for additional experiments.

### Cell Culture and Treatment

The human adenocarcinoma lung epithelial type II cell line A549 was cultured in DMEM with 10% FPS (Fetal Bovine Serum) and 1% Penicillin–Streptomycin (P/S) at 37°C and 5% CO_2_. The cells were seeded and treated for 8 h with different concentration of FPS-ZM1 (500–1000 nM) and then added 1–3% CSE with FPS-ZM1 for 8 h. For experiment of MAPK inhibitor, the cell was pretreated for 2 h with 30 μM SB202190 or SP600125 and then added 1–3% CSE for 8 h. The 10–20 μg/ml S100A6, S100A8 and HMGB1 (High Mobility Group protein B1) was treated for 48 h in the cells. Additionally, the mouse lung epithelial type II cell line MLE12 purchased from American Type Culture Collection (ATC) for single experiment of MAPK inhibitor. MLE12 was cultured in DMEM/F-12 supplemented with 2% FPS, 1% P/S, 2 mM L-glutamine, 10 mM HEPES, 0.005 mg/ml insulin, 0,01 mg/ml transferrin, 30 nM sodium selenite, 10 nM hydrocortisone, and 10 nM β-estradiol at 37°C and 5% CO_2_. The cells were seeded and pretreated for 2 h with 20 μM SB202190 or SP600125 and then added 0.25 or 0.5% CSE for 24 h. The human bronchial epithelial cell line BEAS-2B was cultured in DMEM with 10% FBS and 1% Penicillin–Streptomycin (P/S) at 37°C and 5% CO_2_. The cells were seeded and treated 0.5 or 1.0% CSE for 24 h.

### Immunoblotting

Cell lysate was prepared with RIPA lysis buffer, and the 25 μg of protein were separated on 8–15% SDS-PAGE, electroblotted onto nitrocellulose transfer membrane 0.45 μm. The membranes were blocked with 5% skim milk, probed with primary antibodies and secondary antibodies. Finally, the membranes developed using a pierce^TM^ ECL Western Blotting substrate.

### Enzyme-Linked Immunosorbent Assay (ELISA)

The culture supernatant of cells was subjected to ELISA for determination of IL-6 (interleukin-6), TNF-α (tumor necrosis factor-alpha) and soluble RAGE content and cell lysate was subjected to ELISA for determination of membrane-bounded RAGE using a Duoset ELISA development kit according to the manufacturer’s protocol.

### SOD and GSH Activity Assay

The superoxide dismutase (SOD) activity was measured using a commercial SOD determination kit, and the assay for quantitative determination of glutathione (GSH)/glutathione disulfide (GSSG) levels were measured as previously described (Rahman et al., 2006; Lee et al., 2016).

### Measurement of Reactive Oxygen and Nitrogen Species (RONS)

The intracellular reactive oxygen species (ROS) were detected with the peroxide-sensitive cell permeable 2′,7′-dichlorodihydrofluorescein-diacetate (DCF-DA). A549 cells were treated for 8 h with FPS-ZM1 and then loaded with 10 μM DCF-DA for 30 min at 37°C, followed by treated CSE for 8 h. The cells were dissociated with 0.25% trypsin and analyzed by Accuri-C6 flow cytometry at in FL-1 and. For DCF-DA fluorescence staining, the cells were grown on round coverslips, fixed in 4% paraformaldehyde and observed under a confocal laser-scanning microscope. The level of nitric oxide (NO), reactive nitrogen species (RNS) was determined by modified Griess method using the culture supernatant (Lee et al., 2014). The absorbance at 540 nm was measured using microplate reader within 15 min.

### Immunofluorescence Staining

A549 cells seeded on round coverslips were treated with FPS-ZM1 and/or CSE, and fixed in 4% paraformaldehyde, permeabilized with 1% Triton X-100, blocked with 10% normal goat serum and stained with anti-RAGE and anti-Nrf2 in combination with Alex Fluor 488 rabbit anti-goat and Fluor 594 goat anti-rabbit secondary antibody, respectively. Finally, the coverslips were mounted with DAPI to labeling nuclei. The immunostained cells were analyzed under a confocal laser-scanning microscope.

### Statistical Analysis

All experiments were performed at least three times. The statistical analysis of ELISA or band densitometry calculated by ImageJ were conducted using GraphPad Prism 5 software. Each result was tested using a one- or two-way ANOVA followed by Bonferroni’s Multiple Comparison Test and shown as means ± SE. A *p*-value was calculated, and minimum statistical significance was accepted at *p* < 0.05.

## Results

### CSE Promotes RONS Stress and the Release of Pro-inflammatory Cytokines, and Impairs the Cellular Antioxidant Defense System

**FIGURE 1:**
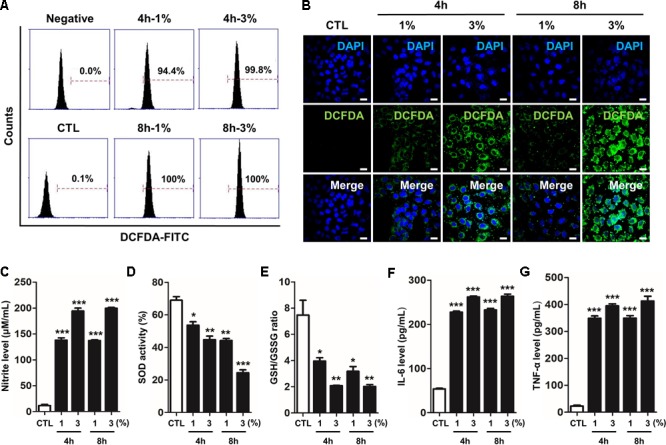
CSE-induced intracellular RONS stress and inflammation in human alveolar epithelial cells. The A549 epithelial cells were incubated in the presence (1–3%) or absence of CSE for 8 h, followed by a measurement of the intracellular redox states. **(A)** The fixed quantity of oxidized DCF-DA probe was evaluated by flow cytometry. The *x*-axis indicate the intensity of intracellular DCF-DA fluorescence, and the *y*-axis represent the mean number of live cells. **(B)** The cells exposed to CSE were loaded with DCF-DA for 30 min before being imaged using confocal laser scanning microscopy. A amount of DCF-DA is linked to live cells damaged by ROS. **(C)** The concentration of nitrite released into the culture medium was determined using the Griess assay 4 h or 8 h after CSE exposure. **(D)** Total SOD activity was measured in cell lysate exposed to CSE. **(E)** The ratio of glutathione (GSH)/oxidized glutathione (GSSG) was detected at different reaction time using a colorimetric assay at OD_412nm_. The calculated the total GSH and GSSG level of test samples based on the standard curve of ∆OD_412nm_/min verses GSH or GSSG standard solutions. A decreased ratio of GSH-to-GSSG is an indication of oxidative stress. The level of **(F)** IL-6 and **(G)** TNF-α were determined by ELISA in culture supernatants. The results are represented by average value of data (±SE). CTL, no treatment. ^∗^*p* < 0.05, ^∗∗^*p* < 0.01, and ^∗∗∗^*p* < 0.001 vs. CTL.

As unrestrained high reactive oxygen and nitrogen species (RONS) concentrations with prolonged oxidative stress are responsible for this vicious sequence of events, we determined the effect of FPS-ZM1 in CSE-induced human alveolar epithelial type II cell line A549. The cells were treated with 1% and 3% concentration of CSE for 4 h or 8 h. As shown in **Figures 1A,B**. To confirm the production of ROS like hydrogen peroxide (H2O2) by CSE in A549, cellular ROS was measured by flow cytometry analysis and DCF-DA staining. In the FACS analysis, the fluorescein derivative DCF was oxidized until 100% in 3% CSE exposure compared to the control for 4 h and 8 h (control; 0.1%, versus 1% at 4 h; 94.4%, 3% at 4 h; 100%, 1% at 8 h; 99.8% and 3% at 8 h; 100%) (**Figure 1A**). Also, number of DCF-DA staining cells were significantly increased in dose- and time-dependent manners in response to CSE exposure for 4 and 8 h (**Figure 1B**). These data indicated that CSE induced oxidative damage by generating ROS in A549. The exogenous production of nitrite, a physiological store of NO, was also accumulated by CSE exposure in a dose-dependent manner in cells (**Figure 1C**). SOD and GSH/GSSG ratio known as antioxidant defense and cytoprotective proteins to protect against free radicals was decreased by CSE exposure in a dose- and time-dependent manners (**Figures 1D,E**). Therefore, these results suggest that cigarette smoke is capable of causing an increase in the generation of various RONS radicals, in turn they are capable of initiating and promoting damage in the respiratory cells. To confirm the inflammatory response by CSE on A549, the protein levels of pro-inflammatory cytokine IL-6 and TNF-α were determined by ELISA. CSE significantly increased the release of both IL-6 and TNF-α cytokines in culture supernatants of cell (**Figures 1F,G**). These results suggest that CSE increased RONS by declining the antioxidant enzymes and inflammatory cytokines in A549.

### CSE Induces the Alternations of RAGE Distribution via Nrf2 Nuclear Migration and MAPK Activation in Human Alveolar Type II Epithelial Cells

**FIGURE 2:**
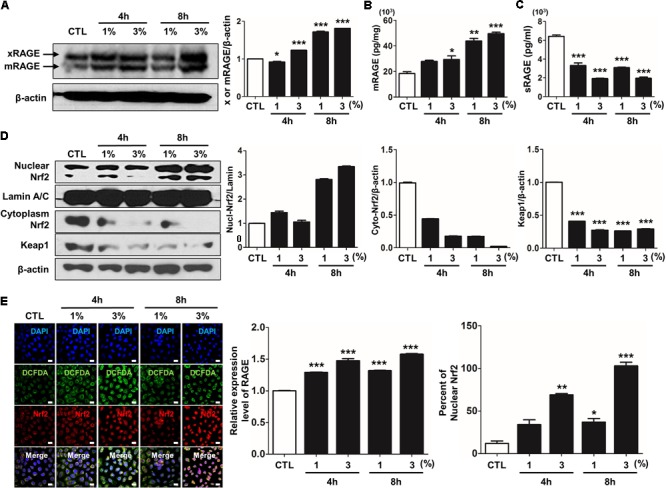
CSE-induced alternations of RAGE distribution through Nrf2 nuclear migration in human alveolar epithelial cells. The expression level of RAGE and Nrf2-related proteins was detected in A549 cells incubated in the presence (1–3%) or absence of CSE. **(A)** Western blotting shows the expression levels of xRAGE and mRAGE, membrane-bound RAGE isoform in cell lysates. Net intensity of RAGE was normalized to β-actin. The levels of **(B)** mRAGE in lung homogenates and **(C)** secreting sRAGE in culture supernatants of cell after CSE exposure were detected by ELISA analysis. The concentrations of proteins were calculated based on each standard curve data. The results are represented by average value of data (±SE). **(D)** The expression of Nrf2 migration from cytoplasm into nuclear location was determined by western blot. The Lamin A/C and β-actin were used for loading control of the nuclear protein and cytoplasmic protein, respectively. **(E)** The confocal microscopy analysis shows the protein expression of RAGE (*green*), the localization of Nrf2 (*red*) and merged images by immunofluorescence staining. Nuclei stained with DAPI (blue). The number of RAGE-positive cells, Nrf2-postivie cells and double-positive cells chosen fields in three randomly at ×600 magnification. The results are represented by average value of data (±SE). CTL, no treatment. ^∗^*p* < 0.05, ^∗∗^*p* < 0.01, and ^∗∗∗^*p* < 0.001 vs. CTL.

**FIGURE 3:**
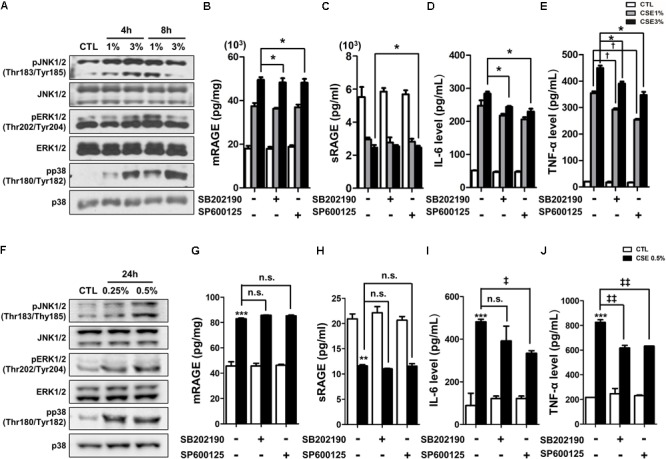
The inhibitory effect of MAPK pathway on CSE-induced alternation of RAGE distribution and inflammation in human alveolar epithelial cells. **(A)** Time and concentration course of MAPK activities in CSE-exposed A549 cells were determined by western blotting of the phosphorylated JNK1/2 (Thr183/Tyr185), ERK1/2 (Thr202/Tyr204), and p38 (Thr180/Tyr182) MAPK. Equal protein loading amounts were proved by β-actin expression. The corresponding density ratio was determined by the average intensity of the bands. The results are represented by average value of data (±SE). ^∗^*p* < 0.05, ^∗∗^*p* < 0.01, and ^∗∗∗^*p* < 0.001 vs. CTL. The A549 cells were pretreated with 30 μM SB202190 or SP600125 for 2 h, and then incubated with 1–3% CSE for 8 h. The levels of **(B)** mRAGE in cell lysate and **(C)** secreting sRAGE in culture supernatants of cell after CSE exposure were detected by ELISA analysis. The protein levels of **(D)** IL-6 and **(E)** TNF-α were examined by ELISA analysis. The MLE12 cells were pretreated with 20 μM SB202190 or SP600125 for 2 h, and then incubated with 0.25% or 0.5% CSE for 24 h. **(F)** The MAPK activities were determined by western blotting, and the level of **(G)** mRAGE in cell lysate, **(H)** secreting sRAGE in culture supernatants of cells and the level of **(I)** IL-6 and **(J)** TNF-α were detected by ELISA analysis. The data showed that MAPK inhibition reversed CSE-induced release of pro-inflammatory cytokine, whereas it failed to reverse abnormal RAGE expression by CSE. The results are represented by average value of data (±SE). ^†^*p*< 0.05, as compared with the cells exposed to 1% CSE alone; ^‡^*p* < 0.05, ^‡‡^*p* < 0.01, as compared with the cells exposed to 0.5% CSE alone. ^∗^*p* < 0.05, as compared with the cells exposed to 3% CSE alone.

Recently, several studies have reported that a functional and susceptibility gene for accelerated COPD or emphysema is RAGE, which is considered as practical biomarker of COPD (Cheng et al., 2013; Yonchuk et al., 2015). The expression of RAGE was assessed by western blot analysis and ELISA. CSE induced the level of increased mRAGE (**Figures 2A,B**) in cell lysate and decreased secreting sRAGE in culture supernatants (**Figure 2C**). To determine whether RAGE expression is associated with the Nrf2 transcription factor regulating a pool of antioxidant and cellular protective genes and DAMP pathway including MAPK signaling on CSE-exerted alveolar type II epithelial cells, we performed western blot and immunofluorescence staining. As shown **Figures 2D,E**, CSE exacted migration of Nrf2 from the cytoplasm into the nucleus and degradation of its cytoplasmic repressor Keap1 accompanied by the phosphorylation of JNK1/2 (Thr183/Tyr185), ERK1/2 (Thr202/Tyr204), and p38 (Thr180/Tyr182) MAPK (**Figures 3A,F**). Then, we asked whether inhibition of MAPK pathway has an effect on CSE-exerted alveolar type II epithelial cell damage. A549 cells and MLE12 were pre-treated with SB202190 (p38 inhibitor) or SP600125 (JNK inhibitor) in the presence of CSE. Pre-treatment with MAPK inhibitors significantly reversed the release of pro-inflammatory cytokines (IL-6 and TNF-α), whereas it failed to recover the expression level of decreased sRAGE and increased mRAGE (**Figures 3B–E,G–J**). These results anticipate MAPK pathway showed to function downstream signal through RAGE and to stimulates the secretion of proinflammatory cytokines, as observed in the pre-treatment profiles of MAPK inhibitors.

### RAGE Antagonist Inhibits RONS Stress and the Release of Pro-inflammatory Cytokines and Rescue Cellular Antioxidant Defense System

**FIGURE 4:**
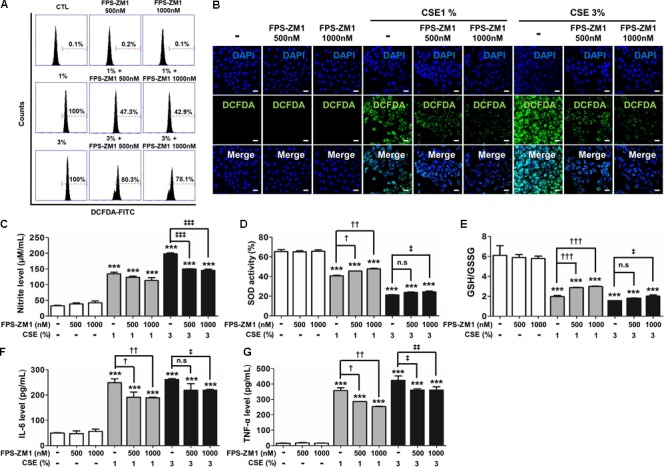
The effect of FPS-ZM1 treatment on CSE-induced intracellular RONS and inflammation in human alveolar epithelial cells. The A549 cells were incubated in the presence of FPS-ZM1 (500–1000 nM) for 24 h, followed by incubation in the presence (1–3%) or absence of CSE for 8 h. The antioxidant effects of FPS-ZM1 in CSE-exposed cells were evaluated by degree of scale of loaded DCF-DA. **(A)** The fixed quantity of oxidated DCF-DA probe was evaluated by fluorescence-activated cell sorting (FACS). The *x*-axis indicate the intensity of intracellular DCF-DA fluorescence, and the *y*-axis represent the mean number of live cells in flow cytometry histograms. **(B)** The cells exposed to CSE and/or FPS-ZM1 were imaged using confocal laser scanning microscopy. Nuclei stained with DAPI (blue). **(C)** The anti-nitration (NO generation) effect of FPS-ZM1 in CSE-exposed cells was indicated by modified Griess reaction in culture supernatants. **(D)** Total SOD activity was measured in cell lysate exposed to CSE and/or FPS-ZM1. **(E)** The ratio of glutathione (GSH)/oxidized glutathione (GSSG) was detected at different reaction time using a colorimetric assay at OD_412nm_. The level of **(F)** IL-6 and **(G)** TNF-α were determined by ELISA in culture supernatants. All figures are representative of three independent experiments, performed in triplicate. Data are expressed as means ± SE. CTL, no treatment; n.s, not significant. ^∗∗∗^*p*< 0.001 vs. CTL; ^†^*p* < 0.05, ^††^*p*< 0.01, and ^†††^*p*< 0.001 vs. 1% CSE treatment; ^‡^*p*< 0.05, ^‡‡^*p* < 0.01, and ^‡‡‡^*p*< 0.001 vs. 3% CSE treatment.

Based on the results of the MAPK inhibitor, we determined whether the specific blockade of RAGE would be directly effective strategy rather than regulates activation of MAKP pathway in CSE-exerted cell damage. As reported in our previous study (Lee H. et al., 2017), we revealed that antagonism of RAGE through FPS-ZM1, a high-affinity novel multimodal and RAGE-specific inhibitor, has effectively suppressed CSE-induced mouse alveolar epithelial cell. In this study, to test whether FPS-ZM1 have such removal capacity of RONS and inflammation apparently remained in human alveolar epithelial cells by CSE exposure for 8 h, A549 cells were exposed to CSE in the presence of 500–1000 nM FPS-ZM1. In DCF-DA flow cytometry analysis and fluorescent staining, 1% or 3% CSE-induced intracellular ROS was reduced in FPS-ZM1 dose-dependent manner (1% vs. 1%+FPS-ZM1 500 nM vs. 1%+FPS-ZM1 1000 nM; 100% vs. 47.3% vs. 42.9%, 3% vs. 3%+FPS-ZM1 500 nM vs. 3%+FPS-ZM1 1000 nM; 100% vs. 80.3% vs. 78.1%) (**Figures 4A,B**). To determine the inhibitory effect of nitrosative/oxidative stress in response to CSE by FPS-ZM1, the levels of endogenous nitrite, SOD activity, and the levels of glutathione (GSH)/oxidized glutathione (GSSG) ratio were assessed in A549 cells. FPS-ZM1 induced reduced the inert end product of NO metabolism and GSH/GSSG ratio in tandem with increased SOD activity (**Figures 4C–E**). Moreover, FPS-ZM1 had an anti-inflammatory effect in line with results from the reduced expression level of IL-6 (**Figure 4F**) and TNF-α (**Figure 4G**). Our data support the notion that blockade of RAGE are able to protect from the consequences of unrestrained RONS and inflammatory response by reducing overall epithelial damage.

### RAGE Antagonist Inhibits the Activation of Redox-Sensitive DAMP Signaling Through Nrf2 in CSE-Induced Human Alveolar Type II Epithelial Cells

**FIGURE 5:**
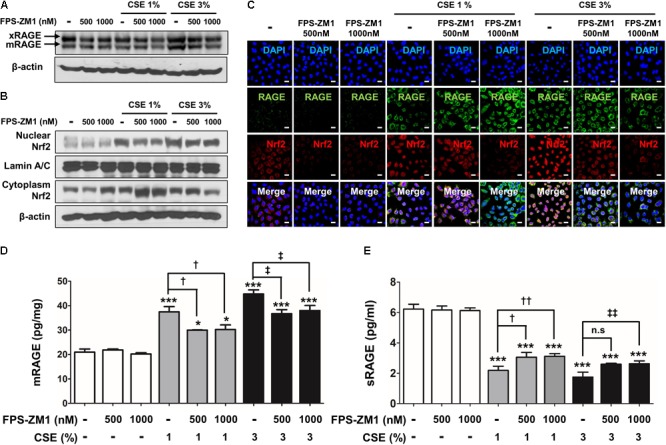
The effect of FPS-ZM1 treatment on CSE-induced alternations of RAGE distribution through Nrf2 nuclear migration in human alveolar epithelial cells. After exposure to CSE for 8 h in the presence or absence of FPS-ZM1 for 24 h, the protein expression level of RAGE and Nrf2 was detected by western blotting, immunofluorescence or ELISA analysis. **(A)** Western blotting shows the expression levels of xRAGE and mRAGE, membrane-bound RAGE isoform in cell lysates. β-actin was used as loading controls. **(B)** The expression of Nrf2 migration from cytoplasm into nuclear location was determined by western blotting. The Lamin A/C and β-actin were used for loading control of the nuclear protein and cytoplasmic protein, respectively. **(C)** For each experimental group, the confocal microscopy analysis shows the protein expression of RAGE (*green*), the localization of Nrf2 (*red*) and merged images by immunofluorescence staining (magnification, ×600). DAPI is in *blue*. The quantitative analysis of **(D)** mRAGE in lung homogenates and **(E)** secreting sRAGE in culture supernatants of cell after CSE exposure were detected by ELISA analysis. Values are presented as the mean ± SE of three independent experiments performed in triplicate. CTL, no treatment; n.s, not significant. ^∗^*p*< 0.05 and ^∗∗∗^*p*< 0.001 vs. CTL; ^†^*p*< 0.05 and ^††^*p*< 0.01 vs. 1% CSE treatment; ^‡^*p*< 0.05 and ^‡‡^*p*< 0.01 vs. 3% CSE treatment.

**FIGURE 6:**
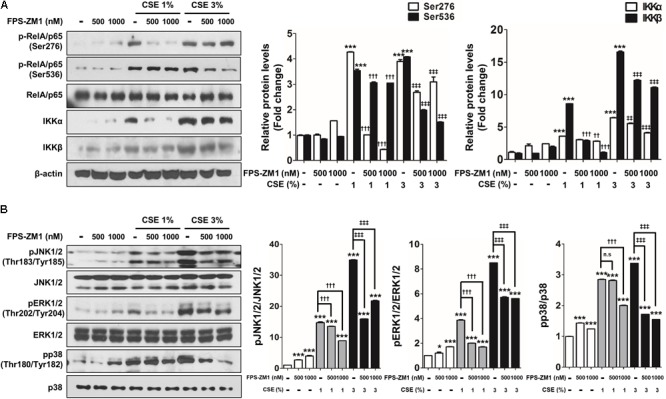
The effect of FPS-ZM1 treatment on CSE-induced activation of DAMP signaling in human alveolar epithelial cells. After exposure to CSE for 8 h in the presence or absence of FPS-ZM1 for 24 h, western blot analysis was performed in cell lysates to confirm the reverse effects of FPS-ZM1 on CSE-induced activation DAMP signaling. **(A)** Protein lysates of isolated A549 cells were subjected to western blotting with either anti-p-RelA/p65 (Ser276), anti-p-RelA/p65 (Ser536), anti-IKKα, or anti-IKKβ antibodies for the assessment of NF-κB activity. RelA/p65 and β-actin were visualized as a loading control. **(B)** Equal protein lysates were subjected to western blotting with either anti-pJNK1/2 (Thr183/Tyr185), anti-pERK1/2 (Thr202/Tyr204), or anti-pp38 (Thr180/Tyr182) antibodies for the assessment of MAPK activity. JNK1/2, ERK1/2, and p38 were visualized as a loading control. The corresponding density ratio was determined by the average intensity of the bands. All values are average ± SE. CTL, no treatment; n.s, not significant. ^∗^*p*< 0.05 and ^∗∗∗^*p*< 0.001 vs. CTL; ^††^*p* < 0.01 and ^†††^*p*< 0.001 vs. 1% CSE treatment; ^‡‡^*p*< 0.01 and ^‡‡‡^*p*< 0.001 vs. 3% CSE treatment.

To determine for the protective, possibly anti-oxidant and anti-inflammatory mechanism of FPS-ZM1 in CSE-induced human alveolar type II epithelial cells, the expression of RAGE and anti-oxidant transcriptional factor Nrf2 was quantified by western blot analysis in A549 cells that had been cultured with CSE and FPS-ZM1. The majority of A549 cells displayed inhibition of mRAGE expression (**Figure 5A** and Supplementary Figure 2A) and migration of cytoplasmic Nrf2 into nucleus after 8 h of culture as depicted in the western blotting and confocal image (**Figures 5B,C** and Supplementary Figures 2B,C). In ELISA analysis, the expression level of increased mRAGE in cell lysate and decreased sRAGE in culture supernatants were also reversed by FPS-ZM1 treatment (**Figures 5D,E**). Moreover, the blocking of RAGE by FPS-ZM1 protected activation of DAMP signaling including the RelA/p65 NF-κB at serine 276 and 536 residues (Ser276 and Ser536), IKKα, IKKβ, and phosphorylated MPAK containing JNK1/2 (Thr183/Tyr185), ERK1/2 (Thr202/Tyr204) and p38 (Thr180/Tyr182) in CSE-exposed cells (**Figures 6A,B**). These results point to that FPS-ZM1 virtually reverses the recalcitrance RAGE-engaged mechanisms through MAPK and NF-κB.

### RAGE Ligand Induces the Activation of Redox-Sensitive DAMP Signaling Through Nrf2 in CSE-Induced Human Alveolar Type II Epithelial Cells

**FIGURE 7:**
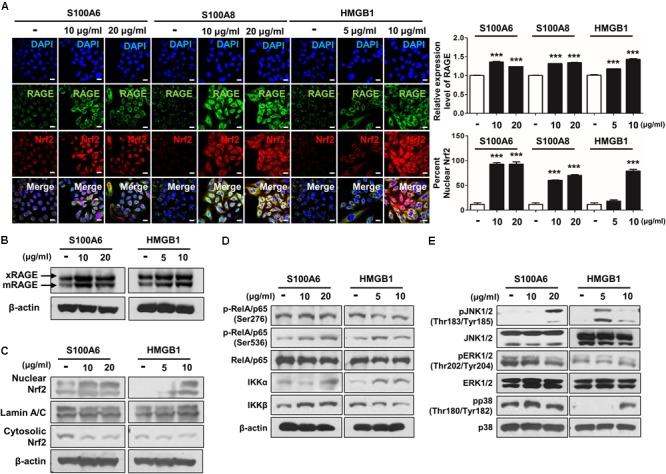
The RAGE/ligand axis-induced activation of RAGE-mediated DAMP through Nrf2 nuclear migration in human alveolar epithelial cells. A549 cells were treated with S100A6 (10–20 μg/ml), S100A8 (10–20 μg/ml) and HMGB1 (5–10 μg/ml) for 72 h. The expression of RAGE and the nuclear migration of Nrf2 were analyzed by immunocytochemistry and western blot. **(A)** The confocal images of RAGE expression and Nrf2 nuclear localization represent under conditions of RAGE ligands treatment. Quantification of RAGE expression was subjected to the number of RAGE-positive cells, and nuclear:cytoplasmic ratio of Nrf2 staining was subjected to densitometric analysis in chosen fields in three randomly at ×600 magnification. All values are average ± SE. ^∗∗∗^*p*< 0.001 vs. no treatment. The protein levels of **(B)** membrane-bound RAGE (xRAGE and mRAGE) and **(C)** Nrf2 nuclear migration were analyzed by western blot. **(D)** The phosphorylation of RelA/p65 at serin 276 (Ser276) and serin 536 (Ser536), IKKα and IKKβ were determined by western blotting with the appropriated antibodies in cell lysate. **(E)** The phosphorylation of JNK1/2 (Thr183/Tyr185), ERK1/2 (Thr202/Tyr204), and p38 (Thr180/Tyr182) MAPKs was detected with the represented antibodies. RelA/p65, β-actin, JNK1/2, ERK1/2, and p38 were used as loading control and the target protein bands were analyzed using ImageJ densitometry. All figures are representative of three independent experiments, performed in triplicate.

To verify whether the accumulation of RAGE ligands directly promote activation of DAMP and Nrf2 excluding the impact of CSE exposure, we treated separately RAGE’s representative ligands including S100A6, S100A8, and HMGB1 in A549 cells for 72 h. As expected, the multiple RAGE ligands excessively induced the expression of mRAGE and Nrf2 nuclear migration in the greater part of cells (**Figures 7A–C**). Moreover, S100A6 and HMGB1 induced phosphorylation of RelA/p65 NF-κB at serine 536 residues (Ser536), IKKα and MPAK pathway containing JNK1/2 and p38 in CSE-exposed cells. However, phosphorylation of RelA/p65 (Ser276), IKKβ and ERK1/2 was not changed by S100A6 and HGMB1 (**Figures 7D,E** and Supplementary Figure 3). These data show extensive RAGE reactivity by elaboration of several RAGE ligands created conditions necessary for the activation of redox-sensitive DAMP signaling such as RAGE downstream, leading to inflammation and cellular damage in parallel with cellular toxicity molecular mechanism occurring in A549 cells by CSE.

## Discussion

Chronic obstructive pulmonary disease/emphysema is characterized by chronic inflammation of the peripheral airways and lung parenchyma, which leads to deprivation of lung function in the wake of progressive narrowing of the airways and shortness of breath. In smokers who develop COPD/emphysema, severely impaired the cellular functions of the alveolar epithelium contribute to irreversible pathological changes (abnormalities of lung surfactant secretion, abnormal inflammatory reaction, oxidative stress and apoptosis) in lung structure and function (Zhao et al., 2010; Padmavathi et al., 2018). Moreover, a diverse isoform of RAGE is selectively expressed in basolateral membranes of ATII cells and well-differentiated ATI cells throughout range of alveologenesis and adult life (Sambamurthy et al., 2015). In adult and pathological states, mouse and human lung express two major RAGE isoforms including a “full-length” membraned-bound isoform (mRAGE) and a soluble isoform (sRAGE). Multiple RAGE isoforms are made through alternative splicing (endogenous secretory receptor for AGE [esRAGE] and/or proteolysis (cRAGE) mediated by proteolytic enzymes. An imbalance between mRAGE and sRAGE have been implicated in programs responsible for acute and chronic inflammation in lung by displaying counteract with each other. Together, this study focused on determining the cellular function of human type II alveolar epithelial cells by CSE and RAGE-related DAMP signaling.

It has been reported substantial studies in our understanding of genetic factors associated with relatively lung functional parameters through genome-wide association studies (GWAS). The study identified polymorphisms in the AGER as being associated correlations with the FEV1/FVC ratio in plasma of individuals with COPD, Cystic fibrosis (CF) and idiopathic pulmonary fibrosis (Young et al., 2011; Iwamoto et al., 2014; Hunt et al., 2016; Tzouvelekis et al., 2016). Cheng DT et al reported that genetic polymorphisms in the AGER locus are associated with systemic sRAGE and lower sRAGE levels have a chance of a biomarker of emphysema severity (Cheng et al., 2013). Likewise, the level of several RAGE ligand such as advanced glycation end products (AGE), HMGB1 and S100 family are increased in COPD and asthma patients and correlate with disease airflow limitation severity and negatively associated with FEV1% (Hou et al., 2011; Wu et al., 2011; Gopal et al., 2014). Moreover, accumulating experimental data using transgenic mice suggests RAGE signaling has been implicated *in vivo* COPD/emphysema. The RAGE knockout mice have shown functionally increased dynamic lung compliance and decreased peak expiratory flow rate accompanied with lower level of elastin expression in lung tissue (Al-Robaiy et al., 2013). On the other hands, conditional RAGE overexpression mice have shown lethal lung hypoplasia and cyto-differentiation of alveolar epithelium (Reynolds et al., 2011b), and subsequently resulted in increased mean linear intercept, apoptosis, decomposition of elastin, and production of matrix metalloproteinases independent of tobacco smoke (Stogsdill et al., 2013). Reynolds et al. (2011b) also have suggested that tobacco smoke-induced RAGE knockout mice had lower levels of proinflammatory molecules, the number of alveolar macrophage as well as a reduced downstream signaling response compared with wild-type mice, and RAGE siRNA inhibited Ras activation and subsequent NF-κB-mediated proinflammatory cytokines (i.e., IL-1β and CCL5) secretion in CSE-exposed in ATI-like R3/1 and ATII-like A549 cells (Reynolds et al., 2011a; Robinson et al., 2012; Stogsdill et al., 2013). Moreover, they reported that RAGE and RAGE-influenced differential expression of TTF-1 and Egr-1 transcription factor by CSE synergistically elaborates positive feedback mechanism of RAGE-mediated signaling in a post-developmental period, and leads to inflammatory response and alveolar regeneration in pulmonary diseases (Reynolds et al., 2008). Furthermore, in this study, we have demonstrated that CSE induces up-regulated mRAGE and down-regulated sRAGE with activation of downstream MAPK pathway including JNK/ERK/p38 and RelA/p65 together with nuclear migration antioxidant-related transcriptional factor Nrf2 nuclear migration in A549 cells.

Until now, COPD is significantly underdiagnosed and often diagnosed late in advancing stage based on lung function check with the help of spirometry. Moreover, the management of COPD with long-acting bronchodilators and corticosteroids does not reduce disease progression, and there are currently no safe and treatments that effectively suppress chronic inflammation in COPD because of resistant or side effects to drugs. Potential antagonist of inflammatory mediators or specific kinase inhibitors for p38, ERK and JNK MAPK have been used as experimental therapeutic strategies in *in vitro* or *in vivo* COPD model. The specific antibody of TNF, is increased in sputum and serum in COPD patients, have toxicity with increased risk of lung cancer and pneumonia in patients with COPD (Rennard et al., 2007; Dentener et al., 2008) IL-6 receptor (IL-6R)-specific antibody is clinical benefit in rheumatoid arthritis who are recalcitrance to TNF-targeted therapy, but no clinical studies have been implemented in COPD (Strand et al., 2012). For targeting oxidative stress, the clinical trial of Nrf2 activators, such as sulforaphane, bardoxolone methyl (CDDO; 1[2-cyano-3,12-dioxooleana-1,9(11)-dien 28-oyl] or dimethyl fumarate (BG-12) is currently a selective drugs that act on the phosphoinositide 3-kinase δ-subunit (PI3Kδ)-histone deacetylase 2 (HDAC2) pathway leading to defective Nrf2 function in COPD lack specificity (Sussan et al., 2009; Mercado et al., 2011; Gold et al., 2012). Several of the kinases including MAPK are activated in COPD patients, which is involved in the activation of manifold inflammatory transcription factors and genes. Therefore, many researchers have attempted therapeutic strategies with inhibitors for p38, ERK, JNK MAPK targeting the common ATP binding site of kinase in COPD. However, a potent inhibitor of the p38 MAPK (losmapimod) have minimal effects on lung function or sputum neutrophils in patients with COPD (Lomas et al., 2012), no clinical studies with inhibitors of the JNK and ERK1/2 have been accomplished in COPD. In line with that, we decide whether inhibition of MAPK pathway has an effect on CSE-exerted human alveolar type II epithelial cell damage. And then, we confirmed the equivalent experiments using mouse-derived normal alveolar type II epithelial cell line (MLE12) to avoid any controversy about human lung adenocarcinoma tissue derived A549 cell. Our results suggested p38 MAPK and JNK inhibitor reduced CSE-induced pro-inflammatory cytokines expression (IL-6 and TNF-α) whereas they fails to control the refractory RAGE expression. This therapeutic strategies comparatively are stable, but they are difficult to achieve specificity to the development of effective anti-inflammatory treatments for cigarette smoke-induced COPD/emphysema. Above all, although levels of many inflammatory cells and mediators are increased in the lungs of COPD patients or experimental COPD model, it is poorly understood how they are linked and the associated mediators. Moreover, there are extensive clinical phenotypes of COPD based on different symptoms, associated comorbidities, precipitating factors or overlapping with asthma, acute respiratory distress syndrome or pulmonary fibrosis. Although there has been an extensive search for biomarkers to predict the COPD including exacerbations, it is lack of biomarkers to predict therapeutic response. In this respect, although yet to be investigated for RAGE, our reports have suggested that RAGE is most probable biomarker of COPD/emphysema.

At present, FPS-ZM1, a high-affinity novel multimodal and RAGE-specific inhibitor, has demonstrated effectively to suppress ligand-dependent inflammation and oxidative stress in chronic neurodegenerative, diabetic complications. Along with this, our data obtained from chemical RAGE-specific antagonist experiments suggests the involvement of MAPKs and RelA/p65 with IKKα and IKKβ in the activation of theses signaling molecules. FPS-ZM1 exclusively bound to precariously increased mRAGE in CSE-exposed pulmonary epithelial cells, which inhibited Nrf2 nuclear migration by blocking DAMP activation. These results point to the contribution of FPS-ZM1 to CSE-induced alveolar epithelial cells damage may help clarify the cellular and molecular mechanisms involved in the pathogenesis of COPD, which is essential for the development of new therapeutic approaches and FPS-ZM1 hold substantial therapeutic promise to counteract epithelial cell damage in conditions with cigarette smoke extract and activated RAGE-related DAMP signaling. Therefore, our data provide a comprehensive deconvolution of lung mesenchyme, which allowed us to identify a critical drug supplement toward epithelial cell regeneration in respiratory diseases.

We acknowledge several limitations of this study. We used here human lung tissue-derived epithelial cell line and type II pneumocyte lung tumor all in one by Giard et al. (1973). In previous studies, A549 cell line has been used in *in vitro* cell systems and toxicology studies because of characteristic features of AT II cells including production of surfactant proteins, synthesis of phospholipids, cytoplasmic lamellar bodies, and apical microvilli (Lee S. et al., 2017; Kim et al., 2018). However, they have important limitation to characterize other *in vitro* lung cell biology because of distinct difference including the architecture, barrier properties, lipid content and transition to ATI-like phenotype from those of primary AT II cells (Lieber et al., 1976; Mason and Williams, 1980; Salmona et al., 1992; Godfrey, 1997). Therefore, it still leads to a question about the suitability of this cell line. To overcome the limitation, we used in alveolar epithelial cell lines (MLE-12) from transgenic mice maintaining the SV40 large T antigen under the control of the human surfactant protein C promoter region in previous and present study. Additionally, we confirmed the RAGE expression and HMGB1, RAGE ligand, in human bronchial epithelial cell line (BEAS-2B). Likewise, RAGE and HMGB1 expression in the bronchial epithelial cell were presented in identical way in A549 and MLE-12 (Supplementary Figure 1). Further studies really should determine the *in vitro* CSE-induced RAGE mechanism and efficacy of RAGE antagonism in COPD with primary alveolar epithelial cell, or RAGE antagonizing with anti-RAGE antibody. Moreover, the *in vitro* cell biology using human primary lung cells from normal subjects and COPD patients would develop approach of drug discovery and selection. In conclusion, we investigated a role of RAGE and Nrf2 in CSE-exposed human pulmonary alveolar type II cell line. CSE exposure induced the alteration of RAGE distribution through redox-sensitive DAMP signaling with Nrf2 nuclear translocation in A549 cells. RAGE blocking using RAGE-specific antagonist FPS-ZM1 effectively exhibited anti-inflammatory and anti-oxidative/nitrosative activity as well as controlled the activation of redox-sensitive DAMP signaling with Nrf2 nuclear translocation by CSE. Given the recognized pleiotropic effects of RAGE antagonist, the strategy targeting RAGE signaling in chronic lung disease could be the efficacious aids to pharmacological research.

## Author Contributions

HL performed the experimental analyses. HL and S-RY designed the study. The results were analyzed by HL, JL, S-HH, and S-RY. IR and S-HH made intellectual contributions to data analysis in the preparation of the manuscript. IR provided supplemental data and edited the manuscript. All authors participated in drafting the manuscript, revised it critically for content, and approved its submission.

## Conflict of Interest Statement

The authors declare that the research was conducted in the absence of any commercial or financial relationships that could be construed as a potential conflict of interest.
