# Supplementary material for: Inhibition of RAGE Attenuates Cigarette Smoke-Induced Lung Epithelial Cell Damage via RAGE-Mediated Nrf2/DAMP Signaling
Source: Front Pharmacol. 2018 Jul 2;9:684. doi: 10.3389/fphar.2018.00684 (PMC6036614; doi:10.3389/fphar.2018.00684)
Supplement: Supplementary file 1 [file Data_Sheet_1.pdf]

Data Supplement

Inhibition of RAGE attenuates cigarette smoke-induced lung epithelial cell damage via RAGE-mediated Nrf2/DAMP signaling

Hanbyeol Lee[1], Jooyeon Lee[1], Seok-Ho Hong[2], Irfan Rahman[3], Se-Ran Yang[1,*]

[1]Department of Thoracic and Cardiovascular Surgery, [2]Department of Internal Medicine, Kangwon National University, 1 Kangwondaehak-gil, Chuncheon, Gangwon 200-701, Republic of Korea, [3]Department of Environmental Medicine, University of Rochester Medical Center, Rochester, NY

Supplementary files

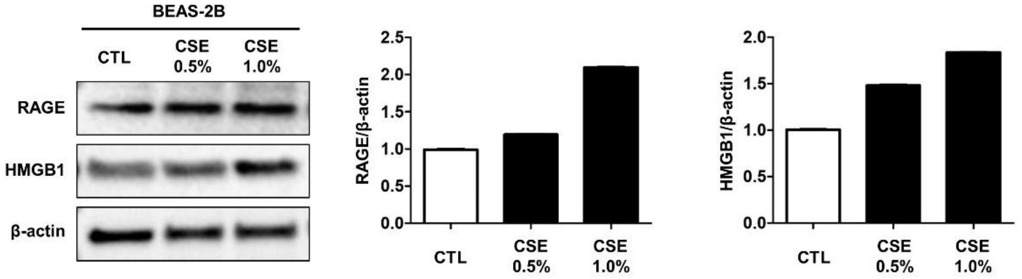

Supplementary Figure 1. The RAGE and HMGB1 were determined by western blotting in 0.5% and 1.0% CSE-exposed BEAS-2B for 24h. Equal protein loading amounts were proved by β-actin. The corresponding density ratio was determined using Image J. CTL, no treatment.

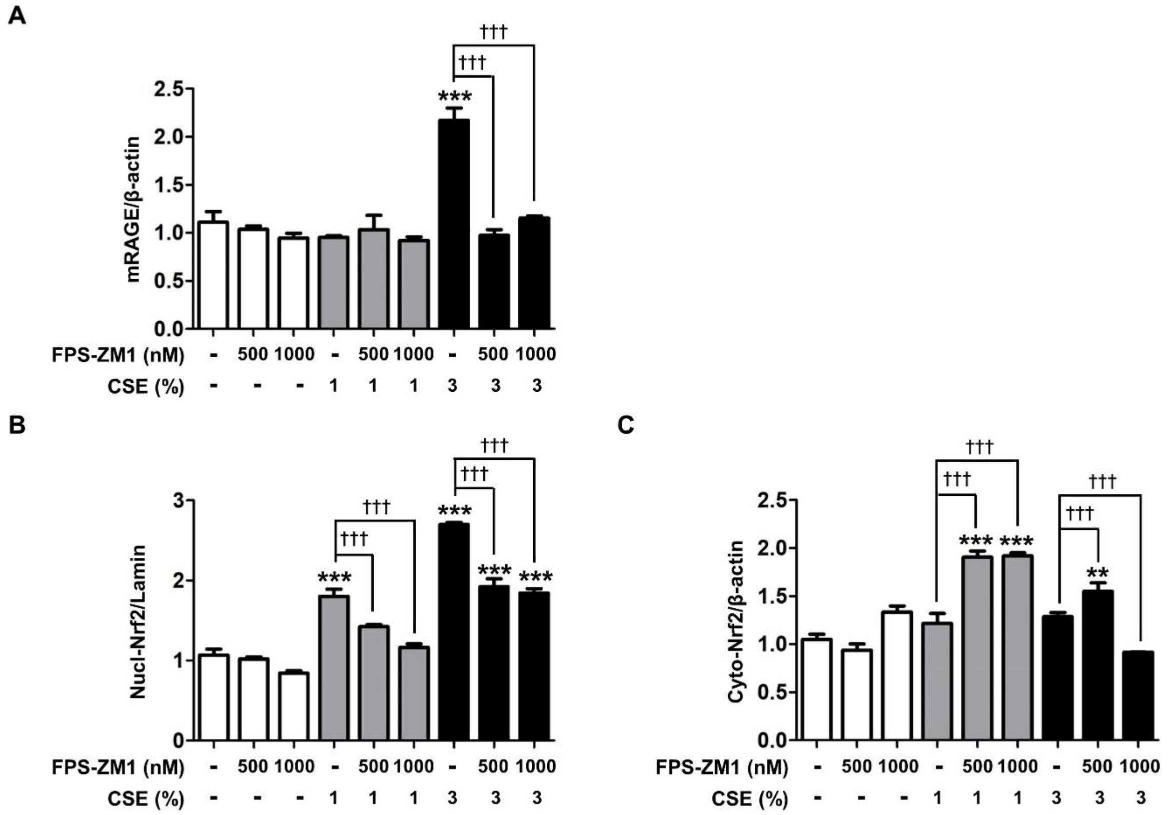

Supplementary Figure 2. A549 cells were treated with CSE in presence of FPS-ZM1 for 8h. The Lamin A/C and β-actin were used for loading control of the nuclear protein and cytoplasmic protein, respectively. Values are presented as the mean ±SE of three independent experiments performed in triplicate. CTL, no treatment; n.s, not significant. ****p<0.001* vs. CTL; †††*p<0.001* vs. 1% CSE treatment; ‡‡‡*p<0.001* vs. 3% CSE treatment. CTL: control.

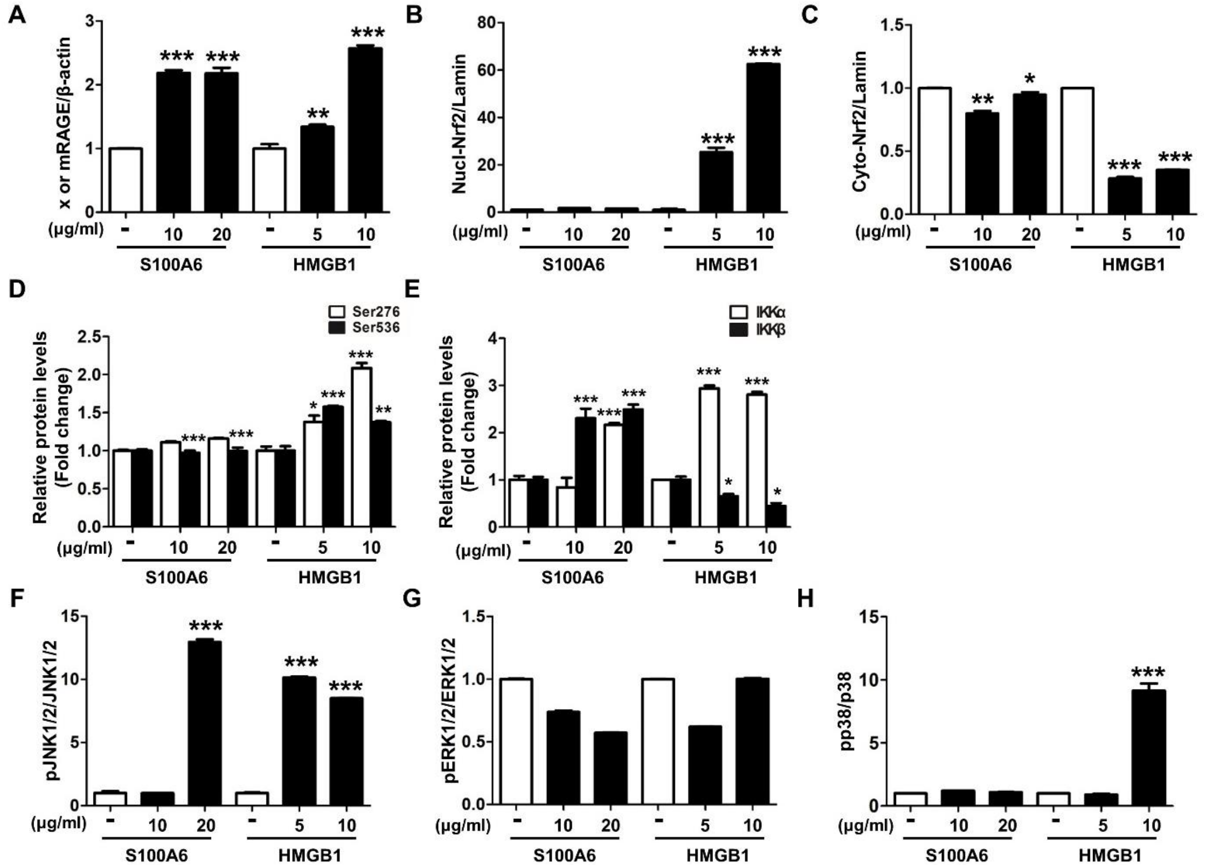

Supplementary Figure 3. A549 cells were treated with S100A6 (10 and 20μg/ml) and HMGB1 (5 and 10 μg/ml) for protein expression of DAMP signaling. The Lamin A/C and β-actin were used for loading control of the nuclear protein and cytoplasmic protein, respectively. Values are presented as the mean ±SE of three independent experiments performed in triplicate. CTL, no treatment; n.s, not significant. *p<0.05 and ****p<0.001* vs. no treatment
